# Transdifferentiation of Human Hair Follicle Mesenchymal Stem Cells into Red Blood Cells by OCT4

**DOI:** 10.1155/2015/389628

**Published:** 2015-02-09

**Authors:** Zhijing Liu, Shi-Jiang Lu, Yan Lu, Xiaohua Tan, Xiaowei Zhang, Minlan Yang, Fuming Zhang, Yulin Li, Chengshi Quan

**Affiliations:** ^1^The Key Laboratory of Pathobiology, Ministry of Education, Department of Pathology, College of Basic Medical Sciences, Jilin University, 126 Xinmin Avenue, Changchun 130021, China; ^2^Advanced Cell Technology, 33 Locke Drive, Marlborough, MA 01752, USA; ^3^Key Laboratory of Biotechnology and Pharmaceutical Engineering, College of Pharmaceutical Sciences, Wenzhou Medical University, Wenzhou 325000, China; ^4^Morphology Center of Hematology Department, Cancer Center, the First Clinical Hospital of Jilin University, Changchun 130000, China

## Abstract

Shortage of red blood cells (RBCs, erythrocytes) can have potentially life-threatening consequences for rare or unusual blood type patients with massive blood loss resulting from various conditions. Erythrocytes have been derived from human pluripotent stem cells (PSCs), but the risk of potential tumorigenicity cannot be ignored, and a majority of these cells produced from PSCs express embryonic *ε*- and fetal *γ*-globins with little or no adult *β*-globin and remain nucleated. Here we report a method to generate erythrocytes from human hair follicle mesenchymal stem cells (hHFMSCs) by enforcing OCT4 gene expression and cytokine stimulation. Cells generated from hHFMSCs expressed mainly the adult *β*-globin chain with minimum level of the fetal *γ*-globin chain. Furthermore, these cells also underwent multiple maturation events and formed enucleated erythrocytes with a biconcave disc shape. Gene expression analyses showed that OCT4 regulated the expression of genes associated with both pluripotency and erythroid development during hHFMSC transdifferentiation toward erythroid cells. These findings show that mature erythrocytes can be generated from adult somatic cells, which may serve as an alternative source of RBCs for potential autologous transfusion.

## 1. Introduction

Blood transfusion is necessary for many patients with emergencies or hematological disorders. However, to date the supply of red blood cells (RBCs, erythrocytes) remains labile and depends on voluntary donations. In addition, transmission of infectious diseases via blood transfusion from unspecified donors remains another risk factor. Therefore, development of safe RBCs, which can be produced from nonimmunoreactive sources and in limitless quantities, would help to address this issue.

Pluripotent stem cells (PSCs) including human embryonic stem cells (hESCs) and induced pluripotent stem cells (iPSCs) have been demonstrated to be one of the best candidates for the generation of RBCs* in vitro*. Primitive erythroid cells as well as erythrocytes with oxygen-carrying capacity have been generated from hESCs using various differentiation systems [1, 2]. hESCs, however, face an ethical issue which may impact their clinical usage. hiPSCs, which are generated by reprogramming somatic cells (e.g., fibroblasts) with defined genes using viral or other vectors [2, 3], overcome this issue. In fact, hiPSCs have been widely used to investigate normal hematopoiesis, pathogenesis, and therapies of hematologic diseases [4–6]. Although recent studies have demonstrated the generation of erythrocytes from hiPSCs using similar approaches as used for hESCs [6, 7], hiPSC-derived erythrocytes exhibited early senescence and expressed little or no adult *β*-globin, and their enucleation was also generally incomplete [8–12]. For example, Lapillonne et al. reported that RBCs generated from hiPSCs via EB formation using a two-step protocol only expressed the fetal-type hemoglobin [13]. Similarly, Dias et al. showed that hiPSC-derived RBCs produced predominately fetal and embryonic hemoglobins, and only mRNA of adult hemoglobin was expressed at a detectable level [9]. Kobar et al. recently reported the switch from fetal to adult hemoglobin after infusion of hiPSC-derived nucleated erythroid precursors into mice [14], which indicates that these cells possess the potential to turn on the expression of *β*-globin gene under appropriate* in vivo* conditions.

Alternative strategies to convert one cell type directly into another cell type by gene manipulation, avoiding the step of reverting to a pluripotent state, have been reported by several groups. Conversions of pancreatic mature cells into functional insulin-producing beta cells by inserting three transcription factors [15] or human fibroblasts into functional endothelial cells, neurons, and keratinocyte-like cells by defined factors have been demonstrated previously [16–18]. Similarly, human mesenchymal cells and fibroblasts were transdifferentiated into hematopoietic cells [19] as well as angioblast-like multipotent progenitor cells or multilineage blood cells [20, 21].

OCT4 (POU5F1) is a key transcription factor for reprogramming and cell type transdifferentiation and also plays an important role in maintaining pluripotency and self-renewal of PSCs [22, 23]. Previous study showed that ectopic expression of OCT4, together with hematopoietic cytokine treatment, converted human fibroblasts into multilineage blood progenitors [21]. Recently, Mitchell et al. reported that transduction of OCT4 conferred fibroblasts “plasticity” to transdifferentiate into three germ layers [24]. We hypothesize that the OCT4 transcription factor and its target genes may play an important role in hematopoiesis. However, it has not been reported whether enforced OCT4 expression will be able to convert other cell types into erythrocytes, such as human hair follicle mesenchymal stem cells (hHFMSCs), which are easily accessible, show no immunogenicity, and could be induced to generate iPSCs as we previously reported [25]. Here, we demonstrate that mature enucleated erythrocytes can be generated from hHFMSCs by enforcing OCT4 expression and stimulation with hematopoietic cytokines.

## 2. Materials and Methods

### 2.1. Isolation of hHFMSCs and Adipogenic and Osteogenic Differentiation

The complete hair follies were plucked out and the root tissues were cut off, and hHFMSCs were isolated according to our previous method [25]. Adipogenic and osteogenic differentiation were examined as previously described [25, 26].

### 2.2. Flow Cytometry

Immunophenotyping of hHFMSCs was carried out using a BD FACSCalibur Cell Sorting System (BD Calibur) as previously described [25] with minor modifications. hHFMSCs were treated with TrypLE and stained with monoclonal antibodies anti-CD44, anti-CD34, and anti-CD166 (1 : 100, BD) in addition to antibodies used in our previous study [25]. hHFMSC^OCT4^ and floating cells were treated with TrypLE. Live cells were identified by 7-amino actinomycin (7AAD) exclusion and analyzed for EGFP expression. To detect the expression of hematopoietic markers, single cells were stained with fluorochrome-conjugated monoclonal antibodies PE-anti-CD45 (1 : 100, BD Pharmingen) and PE-Cy5-anti-CD34 (1 : 100, Cell Signaling Technology). For CD133 detection, Alexa Fluor-555 goat anti-mouse IgG (1 : 200, Cell Signaling Technology) was used as the secondary antibody.

### 2.3. Cell Culture and Differentiation

hHFMSCs and transduced hHFMSCs (hHFMSC^OCT4^) were cultured in H-DMEM/F12 (Gibco) medium supplemented with 10% FBS (Gibco), 100 U/mL penicillin-streptomycin (Hyclone), and 10 ng/mL bFGF (R&D Systems). 293T cells were cultured in H-DMEM (Gibco) supplemented with 10% FBS and 100 U/mL penicillin-streptomycin. hHFMSC^OCT4^ were cultured on Matrigel-coated dishes (cat^#^354277, BD) in hematopoiesis medium (StemSpan SFEM Serum-Free Medium (Stem cell technologies)) supplemented with 10% knockout serum (Gibco), 50 ng/mL BMP4, 50 ng/mL VEGF, 20 ng/mL bFGF, 100 ng/mL SCF, 100 ng/mL Flt3, 20 ng/mL IL3, 20 ng/mL IL6, 20 ng/mL G-CSF, 30 ng/mL IGF-II, 3 U/mL EPO, 100 ng/mL TPO (R&D Systems), and 100 U/mL penicillin-streptomycin for 10–15 days. Cells were then treated with TrypLE (Gibco) and cultured in erythroid cell expansion medium (StemSpan SFEM Serum-Free Medium), supplemented with 0.5% methylcellulose, 10% knockout serum, 100 ng/mL SCF, 20 ng/mL IL3, 3 U/mL EPO, 40 *μ*g/mL myoinositol (Sigma-Aldrich), 10 *μ*g/mL folic acid (Sigma-Aldrich), 203 ng/mL vitamin B12 (Sigma-Aldrich), 160 *μ*M monothioglycerol (Sigma-Aldrich), and 100 U/mL penicillin-streptomycin for 5–10 days. Half-medium was changed every two days. Then erythroid precursors were cultured in erythroid enucleation medium (StemSpan SFEM medium, supplemented with 0.5% methylcellulose, 5% knockout serum, and 100 U/mL penicillin-streptomycin).

### 2.4. Lentivirus Production and Lentivirus Transduction

Lentiviral vector pLV-EF1*α*-OCT4-IRES-EGFP and packaging plasmids expressing gag-pol, pVSVG, and rev genes were obtained from Institute of Biochemistry and Cell Biology of Shanghai Life Science Research Institute, Chinese Academy of Sciences. These vectors were transfected into 293T cells by FuGene HD (Roche). Viral supernatants were harvested at 48 h and 72 h after transfection and concentrated by ultracentrifugation. Viruses were transduced in the presence of 5 mg/mL polybrene.

hHFMSCs (1 × 10^4^/well) were seeded on Matrigel-coated 12-well plate and cells were infected with lentivirus expressing OCT4 in the presence of 5 mg/mL polybrene for 24 h. Then the cells were validated by flow cytometry, RT-PCR, and Western blot.

### 2.5. RT-PCR and qPCR

Total RNA was isolated by Trizol Reagent (Invitrogen). RNA was then subjected to cDNA synthesis using M/MLV kit (TAKARA) and semiquantitative PCR reactions of OCT4 were performed with PCR master Mix (TAKARA). OCT4 target genes related to erythroid development and pluripotency obtained from ChIP-chip dataset by Boyer et al. [27] were analyzed by RT-quantitative PCR (RT-qPCR) during erythropoiesis. RT-qPCR was performed using ABI 7300 real-time PCR system (ABI) and samples were normalized to GAPDH with autoset baseline. qPCR was conducted in triplicates using 25 ng of reverse-transcribed cDNA and 0.2 *μ*M of each primer in a 20 *μ*L final reaction volume containing 1 × SYBR Premix EX Taq (TAKARA). PCR cycling conditions were: one cycle of 95°C for 30 sec followed by 40 cycles of 95°C for 5 sec and 60°C for 1 min followed by a final cycle of 95°C for 15 sec, 60°C for 1 min, and 95°C for 15 sec. Using comparative critical cycle (Ct) method with GAPDH as an endogenous control, the relative expression was calculated as 2^−ΔCt^ and compared between two groups. qPCR primer sequences were provided in Table 1.

### 2.6. Western Blot Analyses

Cells were harvested and lysed in 300 *μ*L ice-cold Radio Immunoprecipitation Assay Lysis Buffer supplemented with 1% phenylmethylsulfonyl fluoride and then centrifuged at 12,000 g at 4°C for 30 min to remove cell debris. Protein concentration was determined by using a BCA Protein Assay Kit (Pierce). Cell lysates were separated on 10% SDS-PAGE and then transferred onto a nitrocellulose membrane (Millipore). The membrane was incubated with 5% nonfat milk (Applichem) at 37°C for 1 h and then incubated with primary antibody OCT4 (1 : 1000, Abcam) at 4°C overnight and 1 : 1,000 diluted secondary antibodies conjugated to horseradish peroxidase for 1 h at room temperature. Membranes were finally stained using an ECL Western blotting system (GE).

### 2.7. Cytospin Preparation, Wright-Giemsa Staining, and Immunofluorescence

Approximately 1,000–2,000 cells were washed twice in cold PBS with 2% FBS and diluted in 400 *μ*L of cold PBS plus 1% FBS. Samples were loaded into wells of Thermo Scientific Cytospin 4 (Thermo Fisher Scientific) and spun at 800 rpm for 5 min. Slides were fixed with either methanol/acetic acid (v/v 3 : 1) for 3 min or precooled acetone for 10 min and dried for 30 min. Slides fixed by methanol/acetic acid were stained with Wright-Giemsa dye for 5 min, followed by soaking in PBS for 10 min and a quick wash in distilled water. Blood cell typing/morphological criteria were confirmed by Morphology Center of Hematology Department, Cancer Center, The First Clinical Hospital of Jilin University.

Slides fixed by acetone were soaked in distilled water for 5 min, followed by rinsing in PBS buffer for 3 times. Slides were then blocked with 10% FBS for 1 h at room temperature and incubated with primary antibodies (optimal working dilutions for all antibodies were listed in Table 2) for 60 min at 37°C in dark. Slides were then washed with PBS buffer for 3 times. Alexa Fluor-555 goat anti-mouse IgG (1 : 200, Cell Signaling Technology) was used as secondary antibody. After washing by PBS, nuclei were counterstained with 5 *μ*g/mL DAPI for 2 min in dark, and cells were visualized with a laser scanning confocal microscope.

### 2.8. Statistical Analysis of Cell Dimensions

The area of cells and nuclei on cytospun Wright-Giemsa-stained slides was measured using Scion Image as previously described [28]. Diameter was calculated from the total cell area, the area of the cytoplasm was calculated as the difference between the total cell area and nuclear area, and then the nuclear-to-cytoplasmic ratio (N/C) was calculated.

### 2.9. Colony-Forming Assay

Cells cultured in hematopoietic medium were disassociated with TrypLE (Gibco) at days 3–5 and analyzed for expression of hematopoietic progenitor markers CD34 and CD45. Total of 10,000 cells were seeded in 1 mL of Methocult H4435 enriched medium (Stem Cell Technologies), and colony-forming units (CFUs) of all hematopoietic lineages (except for megakaryocyte) were scored after 10–14 days of culture using standard morphological criteria.

Megakaryocytic colony-forming assay was detected using the MegaCult-C complete Kit with Cytokines (Stem Cell Technologies) as previously described [21]. CFU-MKs were detectable at days 10 to 14 by staining with MK-specific antigen GPIIb/IIa (CD41).

### 2.10. Statistical Analysis

Data were statistically analyzed by paired Student's *t*-test for comparison of two groups. Differences were considered significant at *P* < 0.05.

## 3. Results

### 3.1. Isolation and Characterization of hHFMSCs

**Figure 1:**
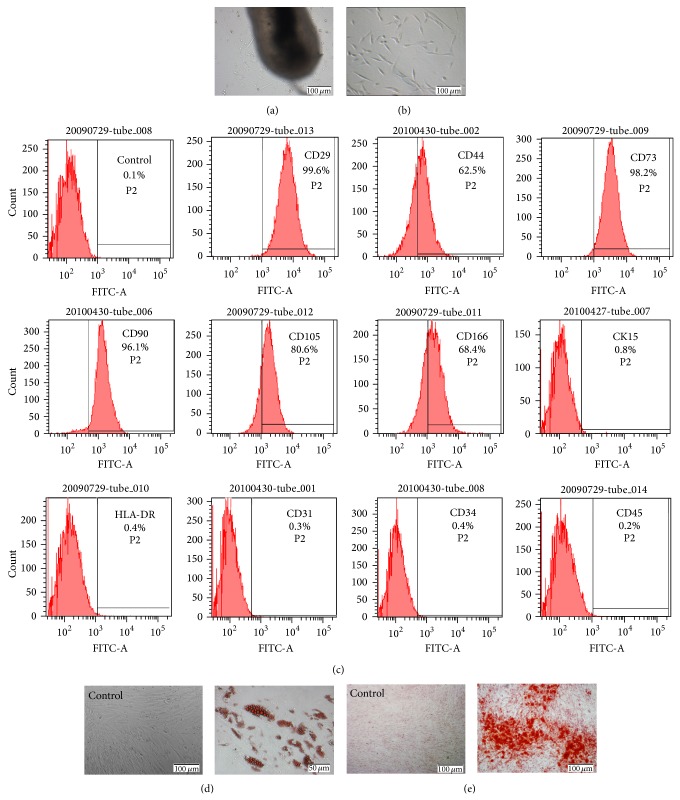
Isolation and characterization of hHFMSCs. (a) The hHFMSCs, resembling typical fibroblast-like cells, migrated out from the hair follicles (original magnification ×100). (b) hHFMSCs from passage 3 (original magnification ×100). (c) Flow cytometric analysis of cell surface markers on hHFMSCs. 2 × 10^5^ cells were incubated with primary antibodies, followed by incubation with a secondary FITC-labeled antibody. Controls were incubated with secondary antibody only. Percentages indicate the fraction of cells that stained positive. (d) Adipogenic differentiation of hHFMSCs. Compared to noninduced control (original magnification ×100), induction after 3 weeks, the number of intracellular lipid droplets was developed and increased and was detected by Oil-red O staining (original magnification ×200). (e) Osteogenic differentiation of hHFMSCs. Calcium nodules were formed after induction for 4 weeks and were demonstrated by Alizarin red S staining (original magnification ×100).

The hHFMSCs, resembling typical fibroblast morphology, migrated out from the hair follicle root tissue and adhered to the surface of the culture plate (Figure 1(a)). The fibroblast-like cells at passage 3 were shown in Figure 1(b).

Flow cytometry analyses showed that the majority of hHFMSCs expressed MSC markers CD29, CD44, CD73, CD90, CD105, and CD166 (99.6%, 62.5%, 98.2%, 96.1%, 80.6%, and 68.4%, resp.). In contrast, hematopoietic (CD31, CD34, and CD45) and keratinocyte (CK15) markers were not detected. Additionally, these cells were negative for the major histocompatibility complex (MHC) class II (HLA-DR) antigen (Figure 1(c)).

Similar to MSCs derived from other sources, hHFMSCs possess multilineage differentiation potential. After adipogenic induction for 3 weeks, the morphology of hHFMSCs changed and a large number of bubble-shaped lipid droplets developed inside these cells. The hHFMSC-derived adipogenic cells contained red lipid droplets in their cytoplasm (Figure 1(d)). When hHFMSCs were cultured in osteogenic inducing medium for 4 weeks, mineralization of osteoblast-like cells was clearly observed by Alizarin red S staining (Figure 1(e)).

### 3.2. Enforced Expression of OCT4 in hHFMSCs Leads to the Changes in Cellular Phenotype

**Figure 2:**
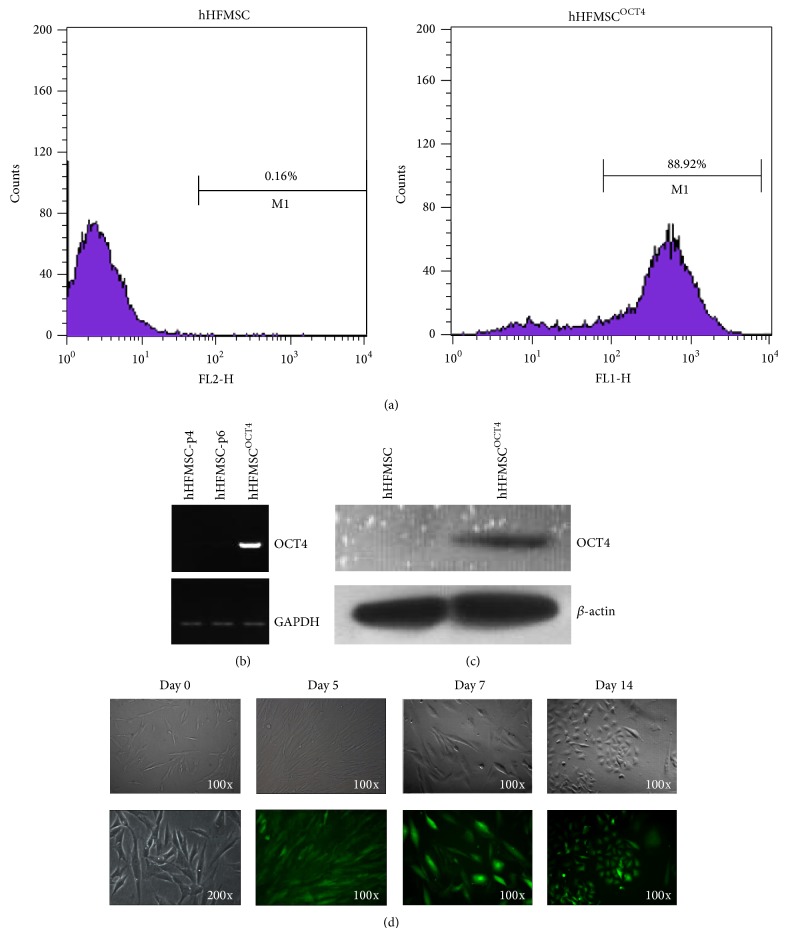
hHFMSCs were transduced with lentivirus encoding OCT4. (a) Flow cytometric plot of GFP expression in hHFMSCs after 12 days of transduction (hHFMSCs^OCT4^). (b) Semiquantitative RT-PCR results for expression of total RNA OCT4 in hHFMSC^OCT4^. (c) Western blot results for expression of total proteins of OCT4 in hHFMSCs^OCT4^. (d) Cell morphologies were changed between 0 and 14 days after OCT4 transduction.

To determine whether hHFMSCs could be converted into erythrocytes directly by enforcing OCT4 expression and hematopoietic cytokine exposure, passage 5 hHFMSCs were first plated on Matrigel-coated dishes and transfected with lentivirus encoding the OCT4 protein and cultured with 10 ng/mL bFGF and 10% FBS. After 12 days, about 90% of OCT4 lentivirus transfected hHFMSCs (hHFMSCs^OCT4^) were GFP^+^ as shown by flow cytometry analysis, suggesting high transduction efficiency (Figure 2(a)). Expression of OCT4 gene was confirmed at both mRNA and protein levels by RT-PCR and Western analyses 12 days after transduction (Figures 2(b) and 2(c)).

Cell morphological change was monitored after transduction. Cell size gradually decreased from day 0 to day 14, and spindle-shaped cells changed to polygonal or round-like cells (Figure 2(d)). After 14 days, a subset of cells emerged from OCT4-transfected hHFMSCs^OCT4^: round-shaped and floating cells were observed when cell density reached about 60% confluence (see Figure S1A-a, b of the Supplementary Material available online at http://dx.doi.org/10.1155/2015/389628). These floating cells were live cells as characterized by trypan blue staining and formed clusters after replating (Figure S1A-c, d), and a population of CD34^+^ cells (up to 2.19%) emerged in these floating cells (Figure S1B), suggesting these cells have characteristics of non-MSCs.

### 3.3. Genenration of Erythroid Cells from Floating hHFMSCs^OCT4^

**Figure 3:**
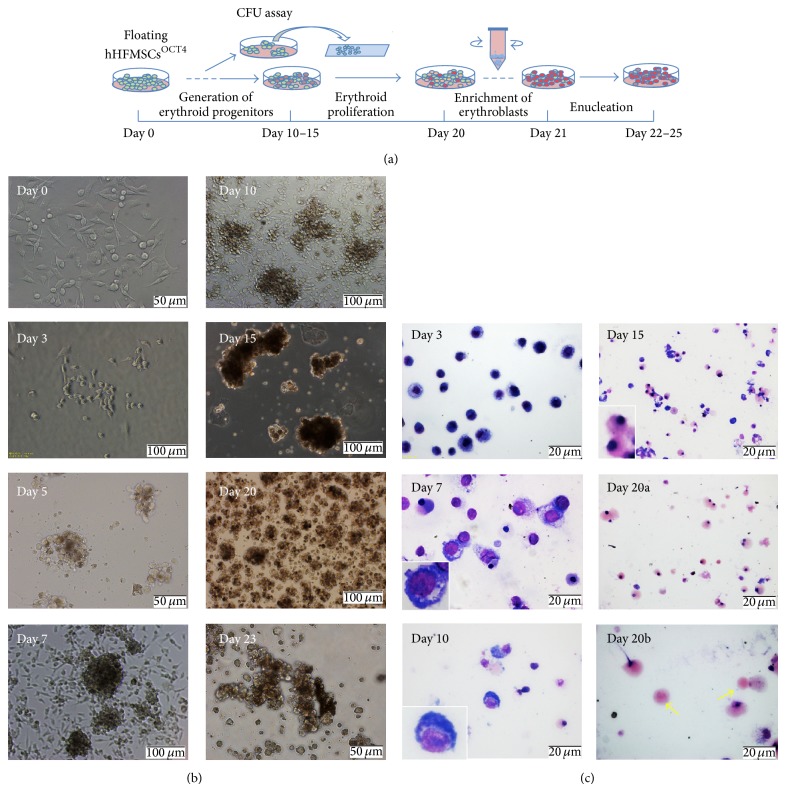
Transdifferentiation of hHFMSCs^OCT4^ into erythrocytes. (a) Schematic of transdifferentiation strategy. (b) Phase-contrast images represented progressive generation of hHFMSC-derived erythroblasts* in vitro* (day 0, day 5, and day 23: original magnification ×200; day 3, day 7, day 10, day 15, and day 20: original magnification ×100). (c) Wright-Giemsa staining detected progressive generation of hHFMSC-derived erythroblasts* in vitro* (day 3, day 7, day 10, and day 20b: original magnification ×400; day 15 and day 20a: original magnification ×200).

In an effort to generate hematopoietic cells and ultimately erythrocytes from floating hHFMSCs^OCT4^, we developed a 4-step protocol (Figure 3(a)). First, floating hHFMSCs^OCT4^ were cultured in hematopoietic medium supplemented with a combination of hematopoietic cytokines (SCF, Flt3-ligand, EPO, and TPO), growth factors, and transferrin chelated iron to generate early erythroid progenitors. Second, early erythroid progenitors obtained after 10 days of differentiation were then induced to proliferate and differentiate into erythrocytes by culturing in erythroid cell expansion medium added with cytokines (EPO, SCF, IL3, and TPO) and nutrition factors that promote erythrocyte development for 5–10 days. Third, cells were collected and diluted in IMDM medium with 0.5% BSA and then centrifuged briefly to remove leukocytes and replated in tissue culture flasks overnight. Late stage erythroid precursors (nonadherent cells) enriched in suspension layer were collected; finally, late stage erythroid precursors were cultured in erythroid enucleation medium to induce maturation and enucleation for 3–5 days (see details in Section 2).

Using this optimized protocol, brown clones emerged between 5 and 10 days after culturing in hematopoietic medium. At day 10, large classic BFU-E, representing the early erythroid progenitors, was observed; late progenitors CFU-E were generated at day 15. Proliferation of CFU-E was reduced after day 15 and discontinued around day 20. Black sediments inside erythrocyte clones were clearly visible at day 23 (Figure 3(b)).

Morphologically, Wright-Giemsa staining showed an obvious change between days 3 and 20. Floating hHFMSCs^OCT4^ showed a strong stained nuclei and basophilic cytoplasm at day 3 after induced differentiation. The first macroblast was observed at day 7, and then basophilic normoblast and acidophilic normoblasts were generated between days 10 and 15. By day 20, a large number of acidophilic normoblasts, some enucleating erythroid cells, and a small number of enucleated erythrocytes were observed (Figure 3(c)). After enrichment, majority of cells are erythroblasts.

**Figure 4:**
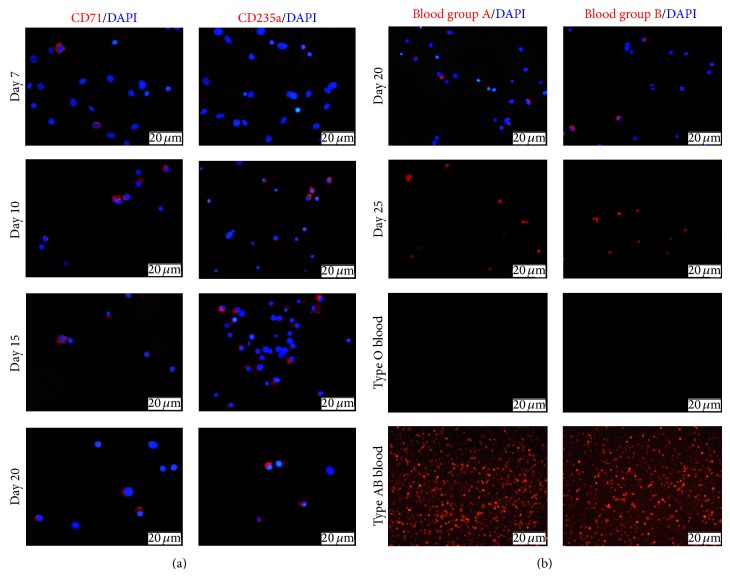
Expression of erythroid markers in hHFMSC-derived erythroid cells. (a) Cell stained with monoclonal antibody against early erythroid progenitor marker CD71 (left) or late erythroid progenitor marker CD235a (right). (b) ABO type characterizations of hHFMSC-derived erythroid cells were detected by staining with monoclonal antibody against A-antigen (left) or B-antigen (right) (original magnification ×200) compared with human type AB blood and type O blood.

Early erythroid marker CD71 (transferrin receptor) was reduced gradually from days 7 to 20, while later erythroid marker CD235a (glycophorin A) was stably increased during this period of time (Figure 4(a)). The fact that both CD71^+^ and CD235a^+^ erythroid cells are detected at the same time implies that erythroid differentiation and development are not synchronized in our culture system. Nevertheless these results indicate that the process of erythropoiesis is recapitulated* in vitro* by our transdifferentiation strategy.

ABO blood group of hHFMSC-derived erythroid cells was also determined by examining the expressions of blood group A and B antigens and compared to human type AB blood and type O blood. These analyses show that hHFMSC-derived erythroid cells are AB type, as both 20 erythroblasts and day 25 enucleated RBCs express both blood group A and B antigens (Figure 4(b)).

### 3.4. Enucleation and Maturation of hHFMSC-Derived Erythroblasts* In Vitro*

To determine whether hHFMSC-derived erythroblasts will be able to mature and generate enucleated erythrocytes* in vitro*, a long-term culture system that keeps these cells healthy is necessary. When hHFMSCs^OCT4^ were cultured in erythroblast expansion medium containing insulin, SCF, EPO, and transferrin chelated iron, but without supplement of nutritional factors (folic acid, monothioglycerol, myoinositol, and vitamin B12), obvious developmental defects occurred in these erythroblasts, which include (1) cells with irregular shapes and partially broken membrane in early erythroblasts, some of them with smaller nucleus posited to poles (Figure S2-a, b); (2) considerable numbers of intermediate erythroblasts with broken membrane (Figure S2-c); (3) big size variation, irregular shape, polarization, and small nucleus in late erythroblasts (Figure S2-d). However, after being supplemented with nutritional factors, cells proliferated and developed into morphologically normal late stage erythroblasts: round-shape and relative uniform size (7–12 *μ*m, Figure S2-e, f).

**Figure 5:**
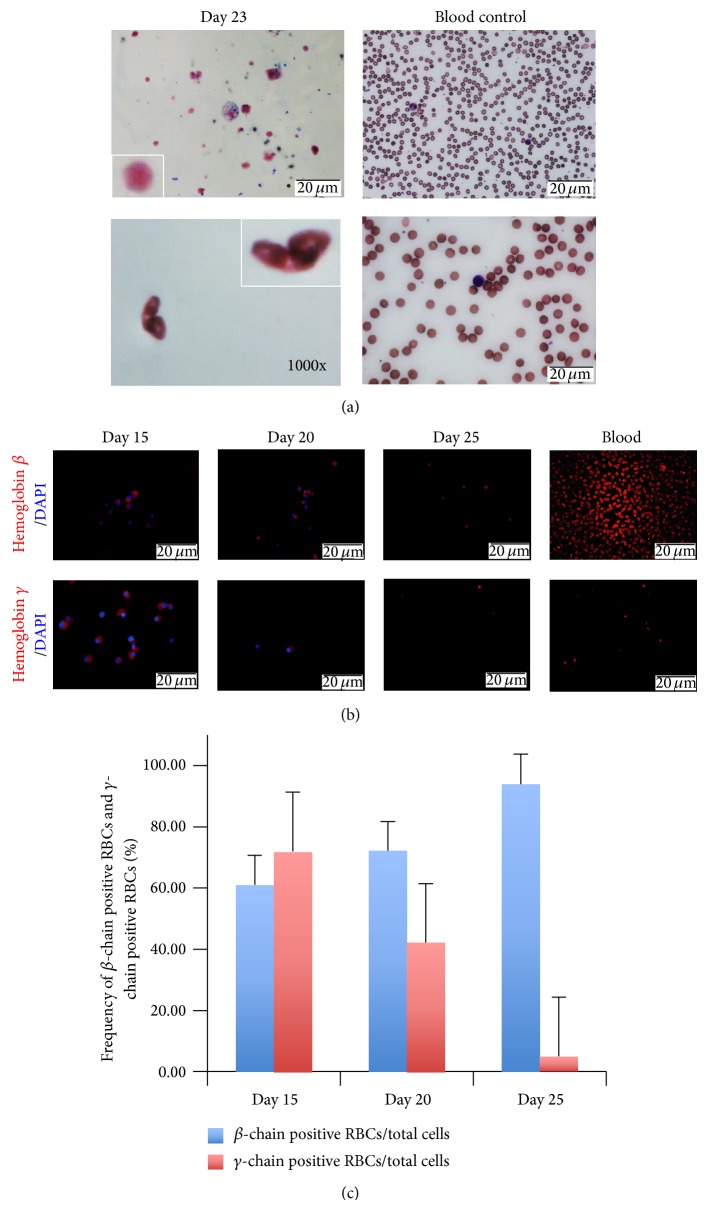
Enucleation and maturation of hHFMSC-derived erythroblasts* in vitro*. (a) hHFMSC-derived erythrocytes were cytospun and stained with Wright-Giemsa dye and compared with red blood cells from human blood. Scale bar represents 20 *μ*m. (b) Cell stained with monoclonal antibody against hemoglobin *β*-chain (left) or *γ*-chain (right). (c) The frequencies of generated RBCs expressing *β*-chain and *γ*-chain hemoglobin were presented by the average ratios of positive cells to total cells from more than 3 fields under microscope.

In addition to the above optimized culture conditions, we observed that it was critical to not remove attached cells (mainly hHFMSC without transduced OCT4) and leukocytes until day 20, when erythroblasts no longer proliferate. Then erythroblasts were cultured in enucleation medium without any cytokines and supplements that resulted in approximately 81% enucleation. The enucleated erythrocytes show similar staining pattern and size as mature RBCs from normal human blood (Figure 5(a)). It was noted that biconcave disc-shaped erythrocytes were also observed (Figure 5(a)), indicating that hHFMSC-derived erythroid cells can mature and enucleate* in vitro* under proper culture conditions which can be improved based on our current system.

Switch of fetal (*α*2*γ*2) to adult hemoglobin (*α*2*β*2) is a key step in maturation of erythrocytes. To determine which type of hemoglobin was expressed in hHFMSC-derived erythrocytes, we used antibodies to specifically detect hemoglobin *β*-chain and *γ*-chain in these cells. At day 15, hHFMSC-derived erythroblasts expressed both *γ*-chain and *β*-chain, indicating that these cells were at early stage of development. From days 15 to 20, the frequency of hHFMSC-derived-RBCs expressing *γ*-chain reduced from 72% to 42%, whereas RBCs expressing *β*-chain increased from 61% to 72% and enucleated RBCs mainly expressed *β*-chain (94%) at day 25, although some enucleated cells also expressed *γ*-chain (5%) which is similar to peripheral RBCs (Figures 5(b) and 5(c)). These results are in contrast to previous reports that erythroid cells derived from hESCs or hiPSCs coexpress high levels of embryonic and fetal globin chains with little or no adult globin [8–13], corresponding to embryonic and early fetal developmental stages, and these results also suggest that hHFMSC-derived erythrocytes are comparable to normal blood adult erythrocytes.

**Figure 6:**
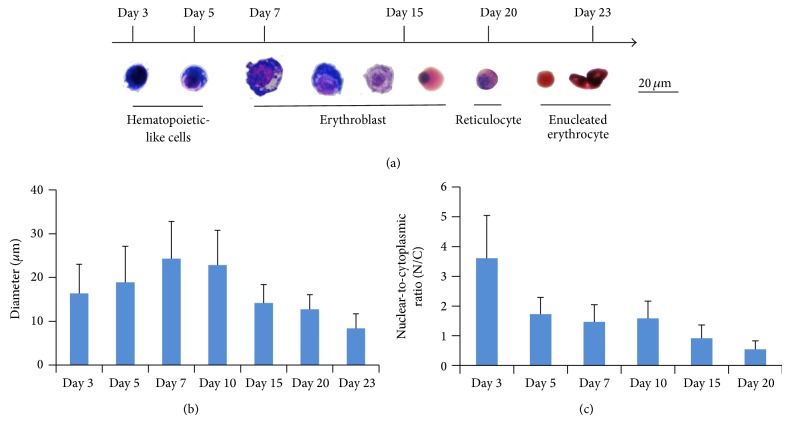
Progressive morphologic changes during generation and maturation of hHFMSC-derived erythroid cells* in vitro*. (a) Progressive morphologic changes from hematopoietic-like cells, erythroblasts to enucleated erythrocytes, and eventually matured erythrocytes are accompanied by significant increase of hemoglobin and decrease in size during their* in vitro* differentiation and maturation. Cells were stained with Wright-Giemsa dye. (b) Diameter decreased with time in culture. Data for each day represent diameters of cells. Enucleated cells decreased to less than half the original diameter on day 3 and were 3 times smaller than the macroblast on day 7. (c) Nuclear-to-cytoplasm ratio decreased with time in culture.

The progressive morphologic changes from hHFMSCs^OCT4^-derived floating cells to enucleated erythrocytes were shown in Figure 6(a). Wright-Giemsa stains showed that maturation of erythrocytes was accompanied by gradual progression from blue to grey-red to pink stain, suggesting an erythroblast (pronormoblast, polychromatic erythroblast, and orthochromatic normoblast) to reticulocyte to mature erythrocyte transition and a progressive accumulation of hemoglobin. We observed that diameter of these cells was slightly increased from 16.35 *µ*m on day 3 to 24.32 *µ*m on day 7 but decreased to 8.4 *µ*m on day 23 (Figure 6(b)), accompanied by progressive decrease of N/C ratio from 1.44 on day 3 to 0.29 on day 20 (Figure 6(c)), indicating substantial nuclear condensation during erythropoiesis.

### 3.5. Direct Transdifferentiation of hHFMSCs into Erythrocytes without Passing through the Stage of Hematopoietic Stem Cells (HSCs)

**Figure 7:**
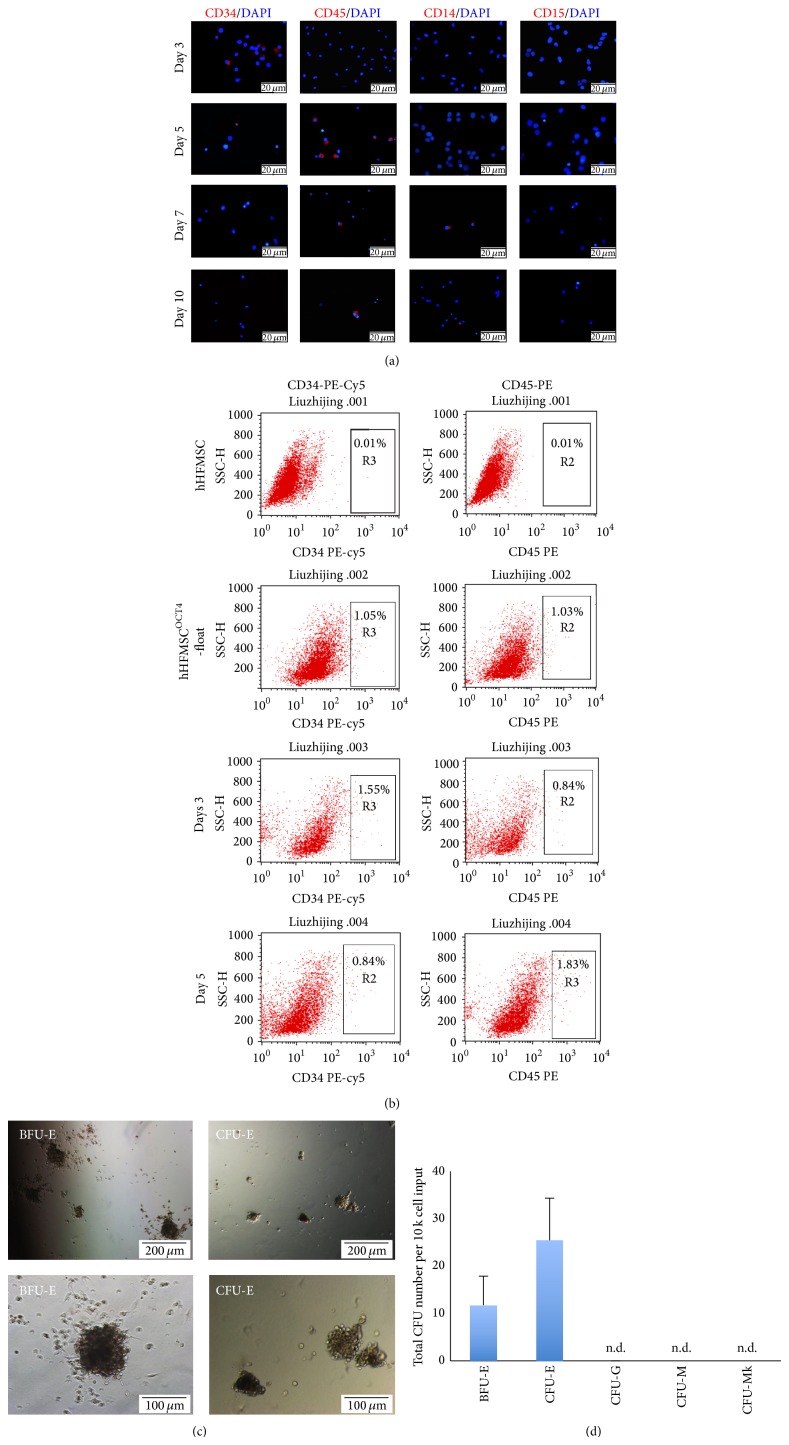
Transdifferentiation of hHFMSCs into erythrocytes without passing by stage of HSC. (a) Cell stained with monoclonal antibody against HSC marker CD34, myeloid progenitor marker CD45, monocyte-macrophage marker CD14, and granulocytic marker CD15. (b) Expression of hematopoietic progenitor markers CD34 and CD45 was detected after hematopoietic stimulation by flow cytometric analysis. On days 3 and 5 after hHFMSC^OCT4^ cells were induced by hematopoietic medium, cells were analyzed by staining with fluorochrome-conjugated monoclonal antibodies PE-anti-CD45 and PE-Cy5-anti-CD34. (c) CFU assay images and (d) quantitative analysis of CFU formation (*n* = 3). Erythroid blast forming units, BFU-E; erythroid CFU, CFU-E; monocytic CFU, CFU-M; granulocytic CFU, CFU-G; megakaryocytic CFU, CFU-Mk. n.d., undetected.

To answer whether hHFMSC transdifferentiation into erythrocytes goes through the stage of hematopoietic stem cell, we collected cells induced for different days and analyzed the expression of HSC marker CD34 and pan-hematopoietic cell marker CD45. Our results showed that CD34^+^ cells were detected from days 3 to 5 and disappeared at day 7, while CD45^+^ cells were emerged at days 5 and 7 (Figure 7(a)). Flow cytometric analyses showed that the percentage of CD34^+^ cells (1.55%) was slightly higher than that of CD45^+^ cells (0.84%) on day 3 but lower (0.84%) than CD45^+^ cells (1.83%) on day 5 (Figure 7(b)). Further analyses showed that a few CD14^+^ monocyte-macrophages were present in day 7 and 10 cultures, but CD15^+^ granulocyte was absent (Figure 7(a)), suggesting that transdifferentiation of hHFMSCs into erythrocytes may be accompanied by a specific lineage leukocyte generation.

The potential of CD34^+^ cells to differentiate into multiple hematopoietic lineages was investigated. Following induction in hematopoietic medium for 3–5 days, hHFMSCs^OCT4^ including CD34^+^ cells were cultured in methylcellulose medium with hematopoietic cytokines for the development of multiple types of hematopoietic colonies. After 10–14 days of incubation, 10^4^ of cells gave rise to, in average, 12 BFU-E, and 25 CFU-E (Figures 7(c) and 7(d)); neither myeloid CFU (CFU-G and CFU-E) nor CD41^+^ megakaryocytic colony (CFU-Mk) was observed. These results suggest that day 3–5 hHFMSCs^OCT4^ including CD34^+^ cells are unipotential specific erythroid progenitors, which do not possess capacity of differentiating into other hematopoietic lineages.

### 3.6. Expression of OCT4 Turns on Erythropoiesis Program in hHFMSCs

**Figure 8:**
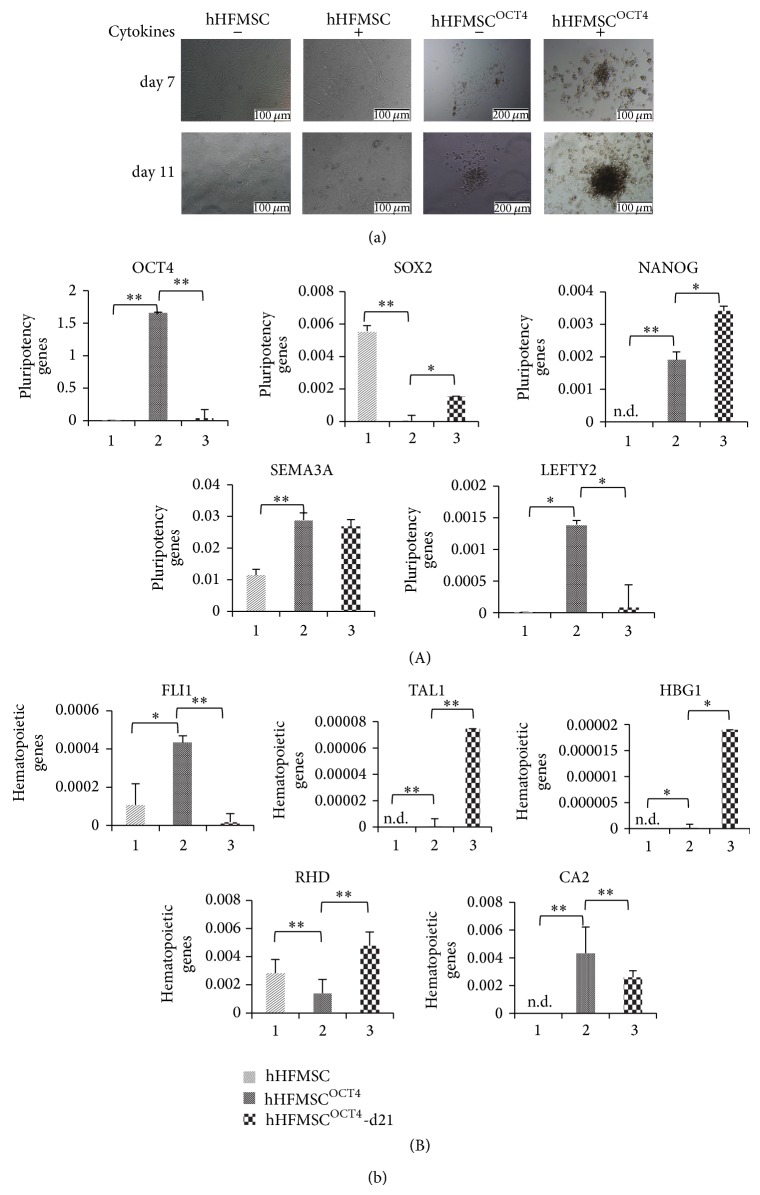
OCT4 gives hHFMSCs a potential for transdifferentiating into erythroid cells by triggering pluripotent state and reexpressing erythroid development genes. (a) OCT4 plays a critical role in transdifferentiation of hHFMSCs into erythroid cells. (b) RT-qPCR detection of OCT4 target genes in transdifferentiation of hHFMSC into erythroid cells. Exogenous OCT4 genes were transduced into hHFMSCs in manner of MOI: 160 (named hHFMSC^OCT4^), and hHFMSCs^OCT4^-d21 were derived from hHFMSC^OCT4^ by hematopoietic cytokines culture for 21 days. (A) Expression of OCT4 target genes associated with pluripotency. (B) Expression of OCT4 target genes associated with erythroid development. ^*^ *P* < 0.05; ^**^ *P* < 0.01; n.d., undetected.

To determine whether OCT4 transduction was required to induce direct conversion of hHFMSCs to erythroid cells, we cultured hHFMSCs in hematopoietic medium using the strategy of step 1 highlighted in Figure 3(a). Results show that hHFMSC themselves do not possess the capability to form hematopoietic cells, even under hematopoietic cytokine stimulation. Although some hHFMSCs treated with hematopoietic cytokines formed capillary-vascular-like structures resembling early endothelial-hematopoietic clusters at day 7, no floating round hematopoietic-like cell was observed, even though brown granules emerged inside some of these cells at day 11. However, hHFMSCs transfected with OCT4 gene generated grape-like colonies and round floating cells without supplementation of hematopoietic cytokines. Furthermore, hematopoietic-like clusters were formed robustly with accumulation of brown granules in these cells, and eventually erythroid clusters emerged on top of attached cells at day 11 after addition of hematopoietic cytokines in the culture (Figure 8(a)), which is consistent with a recent report that direct linage conversion of adult fibroblasts requires OCT4 expression and exposure to reprogramming medium [24]. These results strongly suggest that OCT4 plays a pivotal role in transdifferentiation of hHFMSCs into hematopoietic (erythroid) cells.

As OCT4 plays a critical role on self-renewal and differentiation of PSCs [29, 30], we examined its impact on the expression of genes associated with erythroid development and pluripotency in parental hHFMSCs, hHFMSC^OCT4^, and hHFMSCs^OCT4^-d21 stimulated with hematopoietic cytokines for 21 days. Parental hHFMSCs expressed low levels of neural SOX2 and SEMA3A genes and negligible levels of OCT4, NANOG, and LEFTY2. Enforced expression of OCT4 significantly increased pluripotent gene expression in hHFMSC^OCT4^ (OCT4, *P* = 0.0030; NANOG, *P* = 0.0029; SEMA3A, *P* = 0.0002; LEFTY2, *P* = 0.0322), with the exception of SOX2 gene the expression of which was reduced (*P* = 0.0018). Compared with hHFMSC^OCT4^, expression levels of OCT4 (*P* = 0.0033) and LEFTY (*P* = 0.0366) in hHFMSCs^OCT4^-d21, which were cultured with hematopoietic cytokines for 21 days, were significantly decreased, whereas other two pluripotent genes NANOG (*P* = 0.0469) and SOX2 (*P* = 0.0136) were upregulated, and the expression of SEMA3A kept no change. These results suggest that hematopoietic cytokines may promote specific lineage commitment of hHFMSCs^OCT4^ by balancing the expression of different pluripotent genes (Figure 8(b)-(A)).

On the other hand, hHFMSCs expressed very low to negligible levels of multiple erythroid genes including FLI1 (gene promoting proliferation of erythroid progenitors [31]), TAL1 (a transcription factor for promoting erythroid differentiation [32]), *γ*-hemoglobin gene HBG1, and carbonic anhydrase II gene CA2 (gene promoting the release of oxygen in red blood cells [33]), with the exception of RHD gene which encodes a blood group protein. After enforcing OCT4 expression in hHFMSC^OCT4^, expressions of TAL1 (*P* = 0.0012), CA2 (*P* = 0.0039), HBG1 (*P* = 0.0342), and FLI1 (*P* = 0.0019) were significantly upregulated, but expression level of RHD (*P* = 0.0031) was reduced. After treatment with hematopoietic cytokines, hHFMSC^OCT4^-d21 expressed significant higher levels of TAL1 (*P* = 0.0004), HBG1 (*P* = 0.0407), and RHD (*P* = 0.0055) as compared to control hHFMSC^OCT4^, whereas FLI1 (*P* = 0.0056) and CA2 (*P* = 0.0083) expression were reduced, implying that erythroid development was a complex process that may be regulated by stage-specific expression levels of genes, which were influenced by hematopoietic cytokines treatment (Figure 8(b)-(B)). Indeed, hHFMSC-derived erythroid cells were no longer expandable at day 21, possibly due to a combination of downregulation of FLI1 and upregulation of TAL1, which blocks and promotes erythroid differentiation [31, 32], respectively.

## 4. Discussion

In this study, we demonstrated that enucleated adult type erythrocytes were generated from hHFMSCs by enforcing only one pluripotent factor OCT4 in combination with hematopoietic cytokine treatment. This approach, if the efficiency and scalability can be improved substantially, provides an alternative way to generate adult RBCs for patient-specific transfusion, especially for patients with rare blood types.

hHFMSCs are easily accessible, which provide an alternative stem cell source for patient-specific application. Early studies showed that hair follicle (HF) dermal cells harbored cells with HSC potential that repopulated the hematopoietic system in mice [34]. Similarly, rare but detectable CD45-positive cells were observed in dermal papillae (DP) of human HFs, and CD45-negative DP cells have been found to be able to support the growth of hematopoietic stem/progenitor cells [35]. Recently, human hair follicles were reported as an extrarenal source of erythropoietin (EPO) and a nonhematopoietic target of EPO-R-mediated signaling [36]. Our results show that a small fraction of hHFMSCs is CD45-positive (0.02% of control antibody versus 0.18% of CD45 specific antibody); however hHFMSCs cannot generate hematopoietic cells under the treatment of cytokines (Figure 8(a)), which may be a result of inadequate CD45^+^ cells obtained by plucking the complete hair follicles but not by microdissection of DPs. By enforcing OCT4 expression in these cells, we observed a moderate increase in CD45, CD34, and CD133 positive cells in hHFMSCs (Figure S1B), suggesting that at least the CD34^+^ and CD133^+^ early hematopoietic progenitors are de novel generated from hHFMSCs, especially in the subset of floating hHFMSCs^OCT4^ (>2% of CD34 positive cells). These results, however, will not rule out the possibility that CD34^+^ cells come from CD34^low^ cells residing in hHFMSCs. Although floating hHFMSCs^OCT4^ show hematopoietic-like cell morphologies: round, unattached growth, and forming colonies, hematopoietic clusters were never generated without stimulation of cytokines, which is consistent with the observation that successfully direct conversion of human adult fibroblasts to early hematopoietic and neural progenitor fates requires both OCT4 and short-term exposure to reprogramming media [24]. The true identity of these cells needs to be further investigated.

Another interesting observation is that hHFMSCs^OCT4^ including CD34^+^ cells generated during transdifferentiation are unipotential, only formed hematopoietic BFU-erythroid and CFU-erythroid colonies after replating into methylcellulose medium supporting multiple hematopoietic CFU development; no CFU-G, CFU-M, or CFU of megakaryocyte that shares the same lineage with erythrocyte was developed. This is in contrast to CD34^+^ cells derived from multiple hematopoietic sources including bone marrow, peripheral, and cord bloods, which developed multiple hematopoietic CFUs [37–43]. The CD34 antigen is expressed on both HSCs and committed hematopoietic progenitor cells (such as erythroid and myeloid precursors, common lymphoid progenitors, and natural killer cells) [44] and is also expressed on cell types other than hematopoietic cells such as hemogenic endothelial cells [45]. Animal transplantation studies found that at least a subpopulation of long-term repopulating stem cells is present among CD34 selected bone marrow cells [46]. All evidence suggests that CD34 expression defines a subpopulation with multilineage hematopoietic development potential and the reason behind the lack of multipotential capability of hHFMSCs derived CD34^+^ cells is obscured and needs further investigation.

Erythropoiesis is a multistep process during human development. Primitive erythropoiesis is initiated in the yolk sac with the generation of large nucleated erythroblasts that express mainly embryonic globin chains. Definitive erythropoiesis arises from the fetal liver with the development of smaller enucleated erythrocytes, which express fetal *γ*-globin chain until 6 months after birth and then switch to adult *β*-globin chain [12, 47]. The absence of a nucleus and capability of switching from embryonic (*ε*) and fetal (*γ*) globin chains to adult (*β*) chain are two key features distinguishing definitive from primitive erythroid cells [12]. Specifically the distinct expression patterns of non-*α*-cluster genes corresponding to embryonic, fetal, and adult developmental stages offers a useful measure of erythroid cell maturation stage [1, 28]. Our data clearly demonstrate that hHFMSCs-derived erythroid cells express both fetal and adult globin chains and are capable of enucleation* in vitro*, indicating these cells are most like definitive erythroid cells at fetal/adult development stage. These observations are in contrast to previous studies showing that erythroid cells derived from both hESCs and hiPSCs dominantly express embryonic and fetal but low to negligible levels of the adult globin gene [8–13]. One possibility is that CD34^+^ hematopoietic progenitors are derived from a subpopulation of hHFMSC^OCT4^ cells that are not completely converted into pluripotency and are at fetal/adult development stage. Whereas both human ES and iPS cells are PSCs and their expressions of most, if not all, genes are or have been reset at the embryonic stage, hemangioblasts, CD34^+^ hematopoietic progenitors, and other hematopoietic precursors derived from either human ES or iPS cells lack the intrinsic characteristics and adequate microenvironment to generate definitive mature erythrocytes [8, 13, 48, 49]. This possibility needs further investigation.

In summary, we provided a proof-of-concept that mature erythrocytes could be generated from hHFMSCs, avoiding complete converting adult somatic cells into PSCs. The successful switching of globin chains from embryonic/fetal to adult type plus enucleation of these cells suggests a potential future role for hHFMSCs as an alternative source of red blood cells for transfusion. Red blood cells are the most plentiful cell type in the peripheral blood and are present at a concentration of 5 × 10^12^ cells/liter, which accounts for approximately 40%–45% of the total blood volume [50, 51]. The clinical scale production of red blood cells* in vitro* is an enormous challenge facing all researchers. Although tremendous progress such as various bioreactors has been achieved during past decade [52–54], further investigation will be required to increase the efficiency and scalability of generating red blood cells in abundant supply and in cost-effective ways for clinical application.

## 5. Conclusion

We have developed a robust transdifferentiation strategy* in vitro* which directly converse hHFMSCs into enucleated mature RBCs expressed hemoglobin *β* chain that will provide an alternative way to generate adult RBCs for patient-specific transfusion, especially for patients with rare blood types.

## Supplementary Material

Figure S1. OCT4 transduced hHFMSCs give rise to subsets of hematopoietic-like cells.
Figure S2. hHFMSC-derived erythroblasts kept healthy development by adding nutritional factors.

## Figures and Tables

**Table 1:** qPCR primer sequences.

Gene	Forward primer	Reverse primer
hLEFTY2	GAGGTGCCCGTACTGGACAG	GCCACCTCTCGGAAGCTC
NANOG	ATGGAGGGTGGAGTATGGTTGG	AGGCTGAGGCAGGAGAATGG
SEMA3A	AGTCTGGTGAATAAATGGACAACATTC	GACCTGGCACTGAGCAAATCA
SOX2	TTAGAGCTAGTCTCCAAGCGACGA	CCACAGAGATGGTTCGCCAG
OCT4	CTGAAGCAGAAGAGGATCAC	GACCACATCCTTCTCGAGCC
RHD	GCCTGCATTTGTACGTGAGA	CAAAGAGTGGCAGAGAAAGGA
TAL1	ATGAGATGGAGATTACTGATG	GCCCCGTTCACATTCTGCT
CA2	CAGGGAAGGGTCATACTTGG	GGTACGGCAAACACAACGG
HBG1	ACTTCCTTGGGAGATGCCAC	AAAGCCTATCCTTGAAAGCTCTGA
FLI1	CAGTCGCCTAGCCAACCCTG	GCAATGCCGTGGAAGTCAAAT
GAPDH	CCATGTTCGTCATGGGTGTGA	CATGGACTGTGGTCATGAGT

**Table 2:** Optimal dilutions of antibodies used for immunofluorescence.

Antibodies	Dilution rates
CD34 (Abcam)	1 : 100
PE-CD45 (BD Pharmingen)	1 : 100
CD71 (Abcam)	1 : 200
CD235a (Abcam)	1 : 200
Blood group A antigen (Santa Cruz Biotechnology)	1 : 200c
Blood group B antigen (Santa Cruz Biotechnology)	1 : 200
Hemoglobin *β* (Santa Cruz Biotechnology)	1 : 200
Hemoglobin *γ* (Santa Cruz Biotechnology)	1 : 200
Alexa Flour 555 goat anti-mouse IgG (Cell signaling technology)	1 : 200
CD14 (Abcam)	1 : 100
CD15 (Abcam)	1 : 100
