# Supplementary material for: Transdifferentiation of Human Hair Follicle Mesenchymal Stem Cells into Red Blood Cells by OCT4
Source: Stem Cells Int. 2015 Feb 9;2015:389628. doi: 10.1155/2015/389628 (PMC4337757; doi:10.1155/2015/389628)
Supplement: Supplementary file 1 — Figure S1. OCT4 transduced hHFMSCs give rise to subsets of hematopoietic-like cells. Figure S2. hHFMSC-derived erythroblasts kept healthy development by adding nutritional factors. [file 389628.f1.pdf]

Supplemental materials

Figure S1. OCT4 transduced hHFMSCs give rise to subsets of hematopoietic-like cells.

(A) Emergence of round and floating cells among hHFMSCs^{OCT4}(a, b); phase-contrast image of clones froming after re-attachment of floating cells (c, d). (B) Expression of HSC marked genes CD45, CD34 and CD133 were detected after OCT4 transduction by flow cytometer analysis. hHFMSC ^{OCT4} cells were treated with TrypLE and analyzed for expression of haematopoietic progenitor markers by staining single cells with fluorochrome-conjugated monoclonal antibody PE-anti-CD45 (1:100, BD Pharmingen), for CD34 (1:100) and CD133 antibody (1:50) staining, Alexa Fluor-555 goat anti-mouse and anti-rabbit IgG (1:200, CST) were used as the secondary antibody.

Figure S2. hHFMSC-derived erythroblasts kept healthy development by adding nutritional factors (a, f : original magnification ×400; b, c, d, e: original magnification ×200).

Figure S1

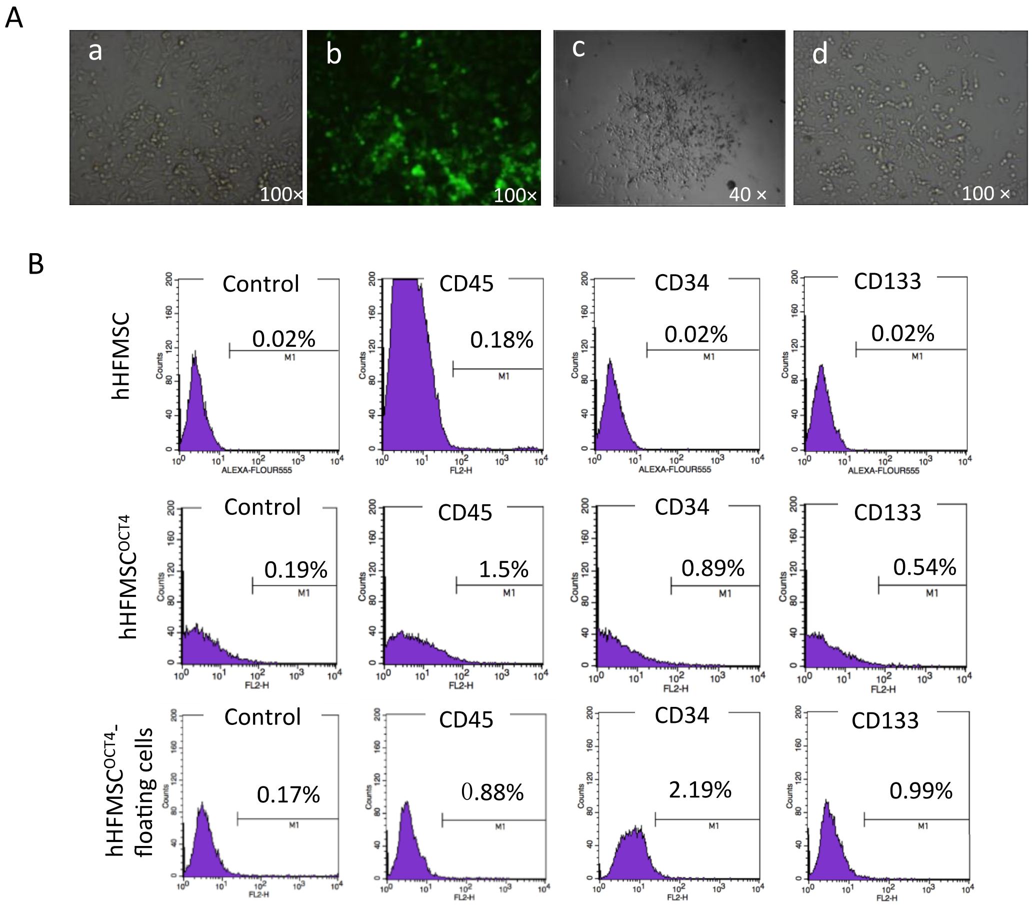
A
a
100×
b
100×
c
40 ×
d
100 ×
B
hHFMSC
Control
0.02%
CD45
0.18%
CD34
0.02%
CD133
0.02%
hHFMSC^OCT4
Control
0.19%
CD45
1.5%
CD34
0.89%
CD133
0.54%
hHFMSC^OCT4-floating cells
Control
0.17%
CD45
0.88%
CD34
2.19%
CD133
0.99%
Counts
M1
ALEXA-FLOUR555
FL2-H

Figure S2

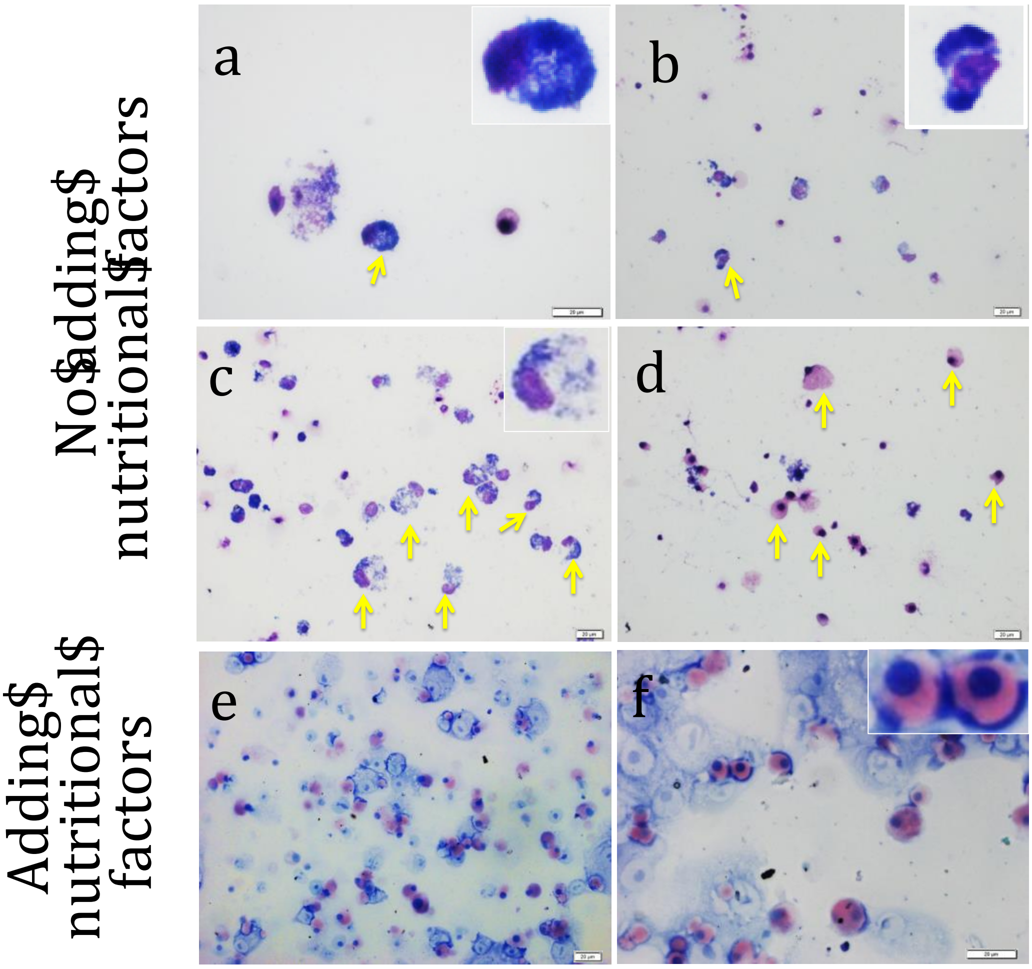

No$adding$ nutritional$factors
Adding$ nutritional$ factors
a
b
c
d
e
f
